# Supplementary material for: PRMT2 links histone H3R8 asymmetric dimethylation to oncogenic activation and tumorigenesis of glioblastoma
Source: Nat Commun. 2018 Oct 31;9:4552. doi: 10.1038/s41467-018-06968-7 (PMC6208368; doi:10.1038/s41467-018-06968-7)
Supplement: Supplementary file 1 — Supplementary Information [file 41467_2018_6968_MOESM1_ESM.pdf]

Supplementary Information (12 Figures, 2 tables) for

PRMT2 links histone H3R8 asymmetric dimethylation to oncogenic activation and tumorigenesis of glioblastoma

Dong.et.al

Supplementary Fig.1

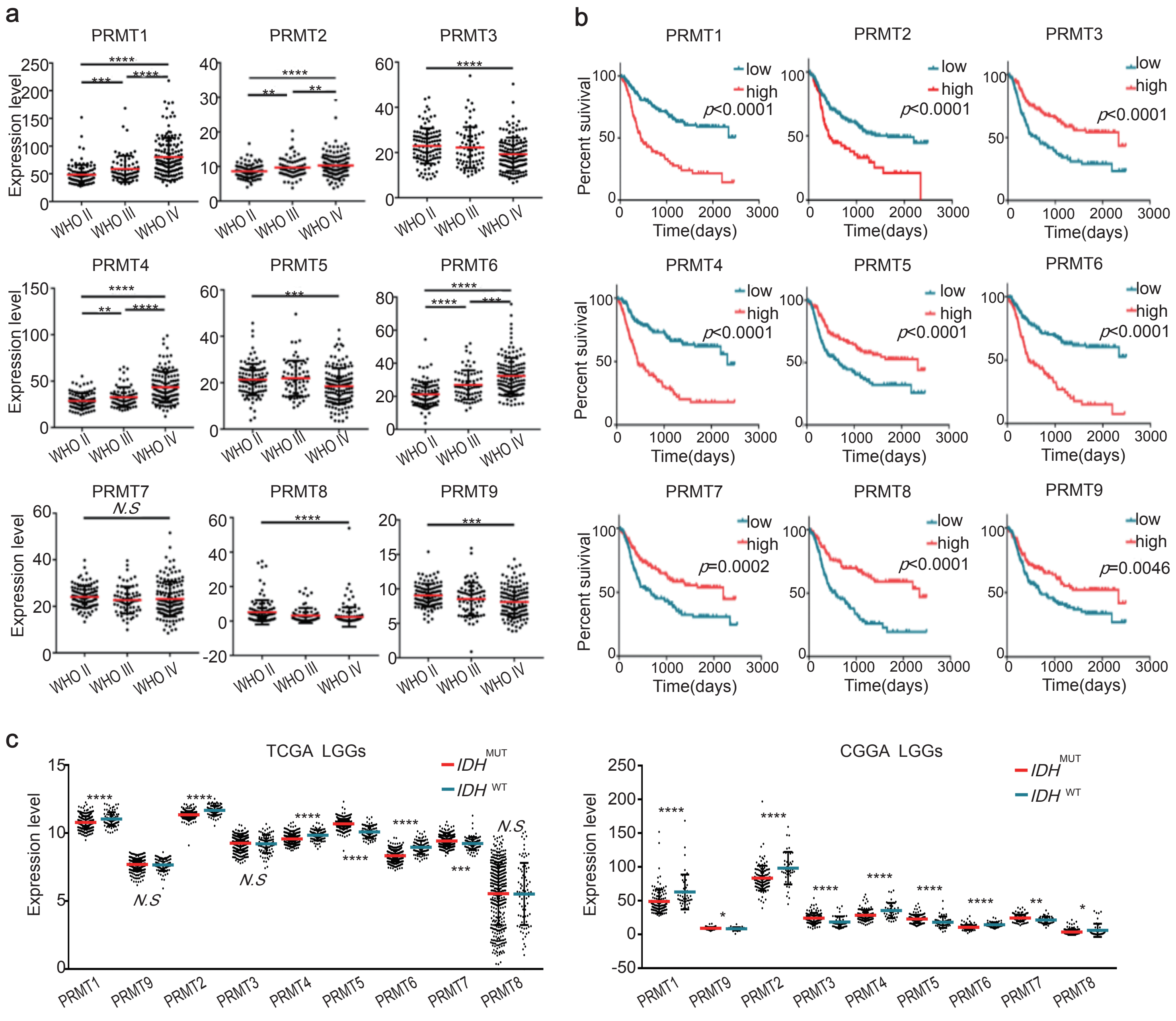

Supplementary Fig. 1 The expression levels of PRMT gene family members in different grade of gliomas and significance in prognosis. (**a**) The expression levels of PRMTs were analyzed in different grade of glioma tissues according to CGGA dataset. Significance level was determined using One-way ANOVA followed by Dunnett's multiple comparisons test. *$p \leq 0.05$,**$p \leq 0.01$,***$p \leq 0.001$ and ****$p \leq 0.0001$. (**b**) Kaplan-Meier survival analyses for correlation between PRMT mRNA expression and survival of glioma patients in the CCGA dataset. (**c**) The mRNA levels of PRMTs were compared between *IDH*^{WT} or *IDH*^{MUT} glioma tissues in TCGA dataset (Left panel) and CGGA dataset (Right panel).

Supplementary Fig.2

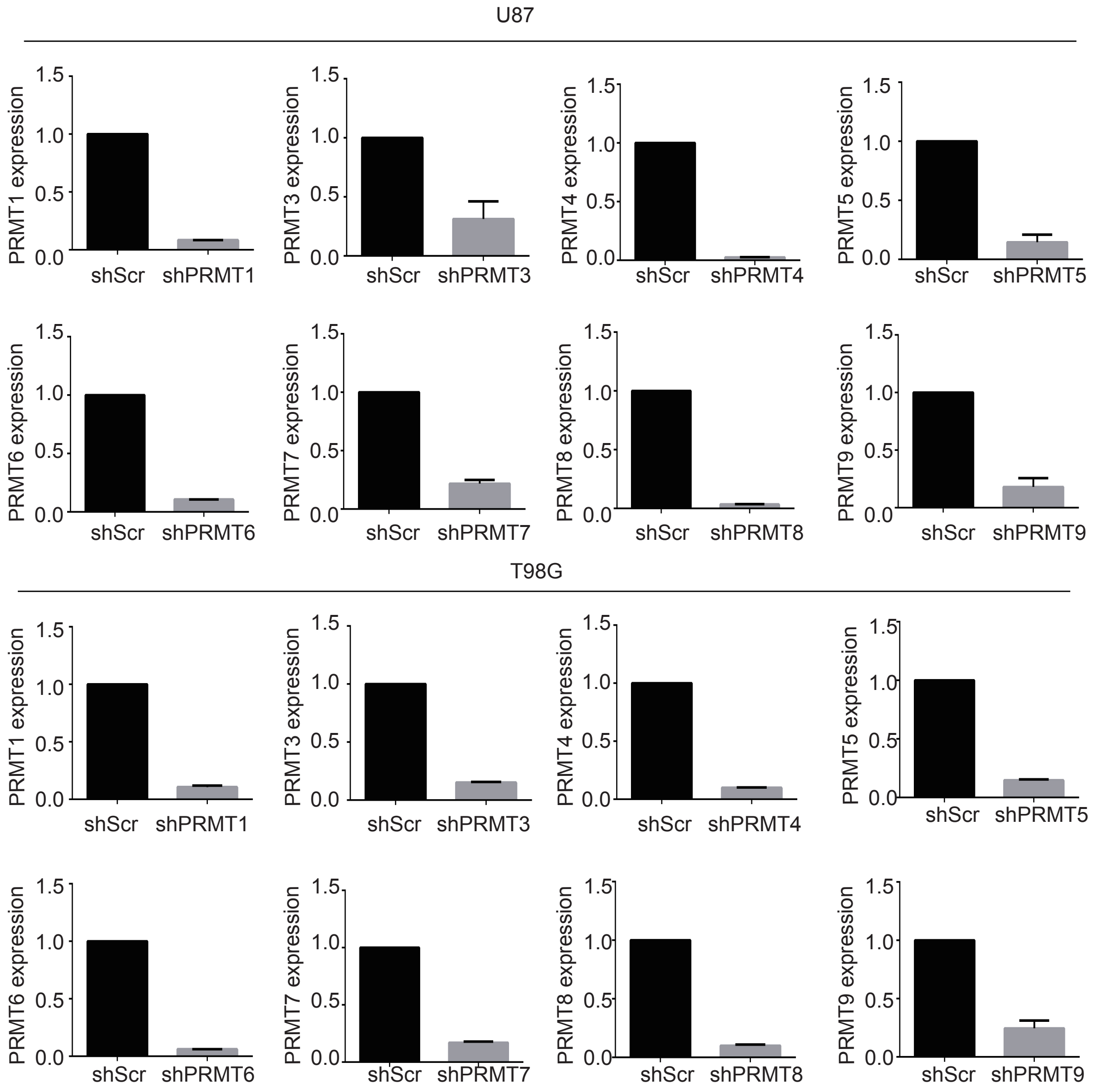

Supplementary Fig. 2 Validation of knockdown effects. qRT-PCR analysis of the knockdown effects for each PRMT gene expression by designated shRNAs in U87 and T98G cells

Supplementary Fig.3

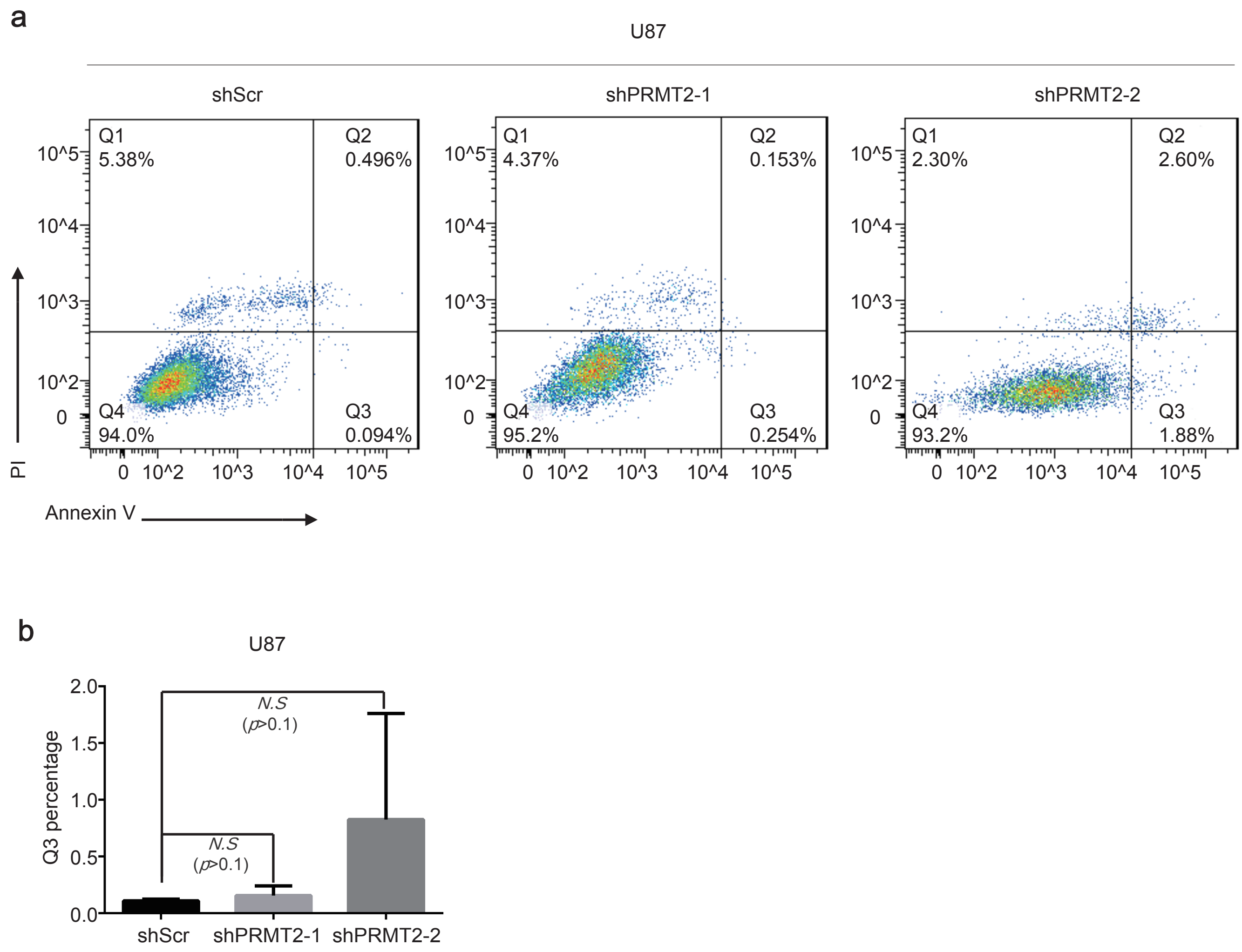

Supplementary Fig. 3 PRMT2 depletion does not result in cell apoptosis. (**a**) U87 cells were treated as indicated and cell apoptosis was assessed by flow cytometry. (**b**) A rigorous statistical analysis by Student's two-sided *t*-tests about apoptosis has been showed.

Supplementary Fig.4

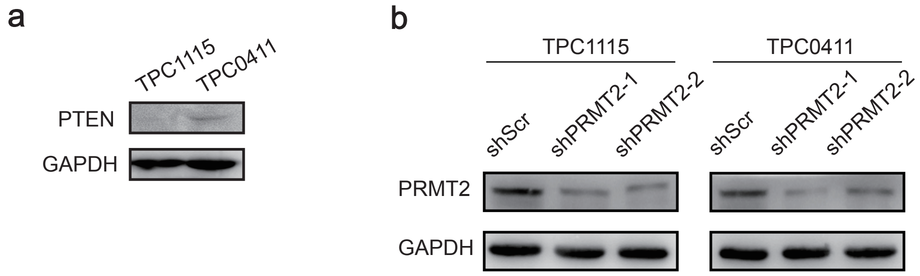

Supplementary Fig. 4 The PTEN status (**a**) and the efficiency of PRMT2 knockdown (**b**) were indicated in TPC1115 and TPC0411 cells through western blot analysis.

Supplementary Fig.5

a

Stem Cell Frequency	U87			T98G		
	shScr	shPRMT2-1	shPRMT2-1	shScr	shPRMT2-1	shPRMT2-1
Lower	1/14	1/103	1/285	1/12	1/87	1/47
Estimate	1/11	1/83	1/212	1/9	1/69	1/39
Upper	1/9	1/67	1/157	1/8	1/55	1/32

Stem Cell Frequency	TPC1115			TPC0411		
	shScr	shPRMT2-1	shPRMT2-1	shScr	shPRMT2-1	shPRMT2-1
Lower	1/11	1/26	1/39	1/12	1/25	1/25
Estimate	1/9	1/22	1/36	1/10	1/20	1/10
Upper	1/8	1/18	1/22	1/8	1/17	1/17

b

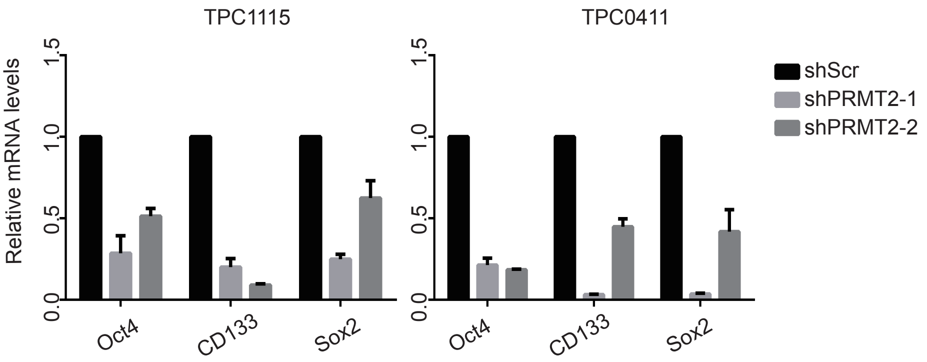

c

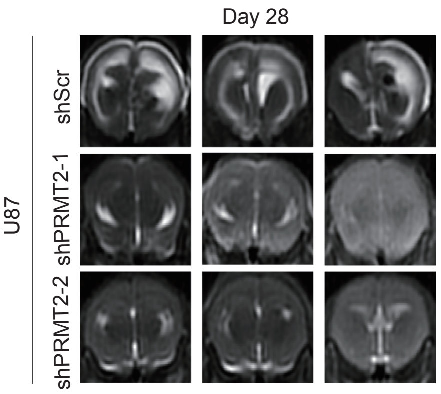

Supplementary Fig. 5 (**a**) Stem cell frequencies from GSCs with or without PRMT2 knockdown were estimated as the ratio 1/x with the upper and lower 95% confidence intervals, where 1 = stem cell and x = all cells. (**b**) The mRNA levels of stem cell markers in TPC1115 and TPC0411 cells with shScr or shPRMT2. Error bars, ± SD, *n*=2. (**c**) MRI scanning of orthotopic tumors. Representative MRI images of brains of immunocompromised mice inoculated with control or PRMT2 knockdown U87 cells at day 28 post tumour implantation.

Supplementary Fig.6

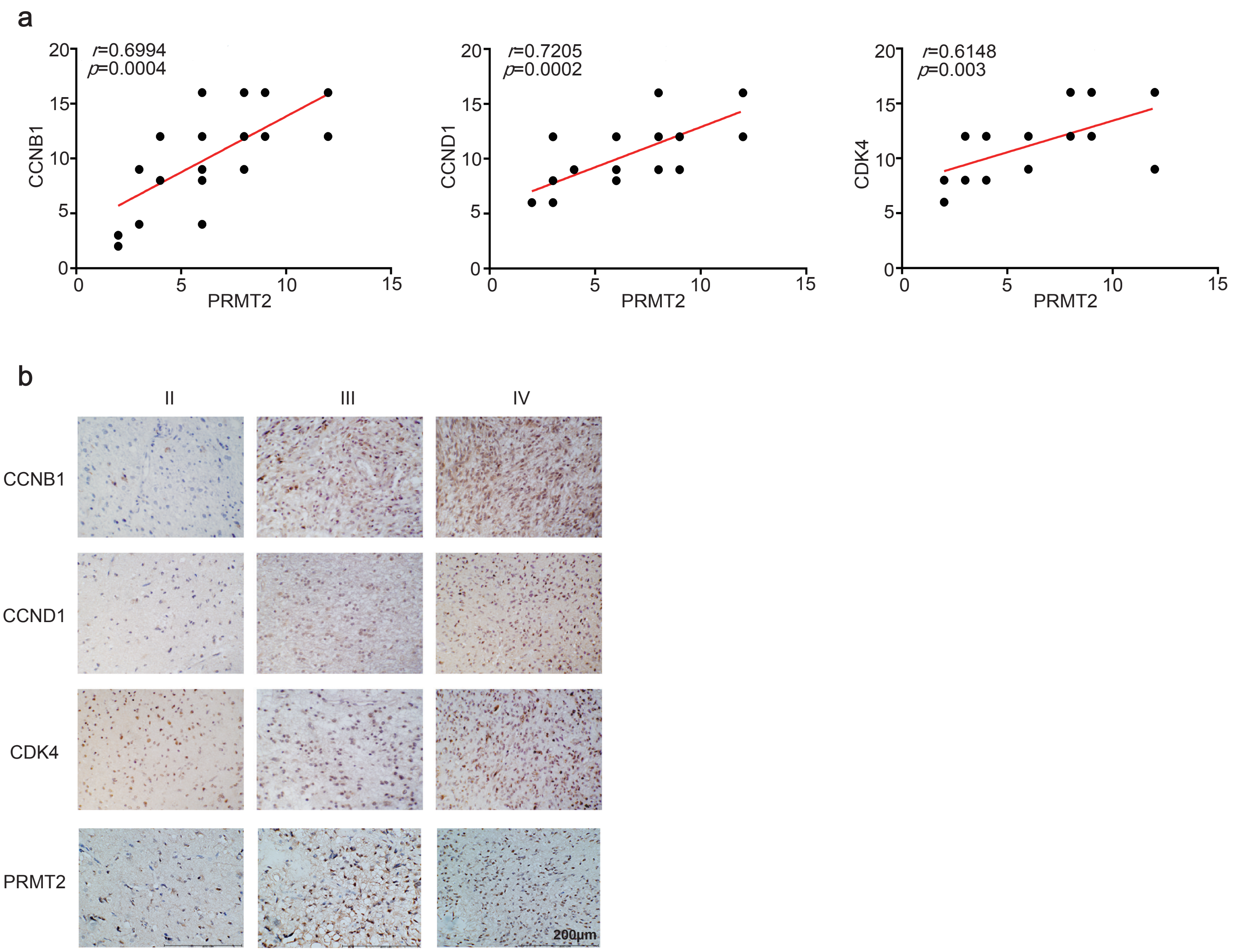

Supplementary Fig. 6 (**a**) Correlation between PRMT2 protein levels with CCNB1,CCND1 and CDK4 protein levels in glioma specimens. Tumor sections from 21 glioma specimens were IHC-stained with anti-CCNB1, CCND1 and CDK4 antibody. The significance of the correlation was determined by Pearson's correlation test. (**b**) Representative examples from IHC analysis of CCNB1, CCND1, CDK4 and PRMT2 protein levels in different grade of glioma specimens are shown. Scale bar, 200 μm.

Supplementary Fig.7

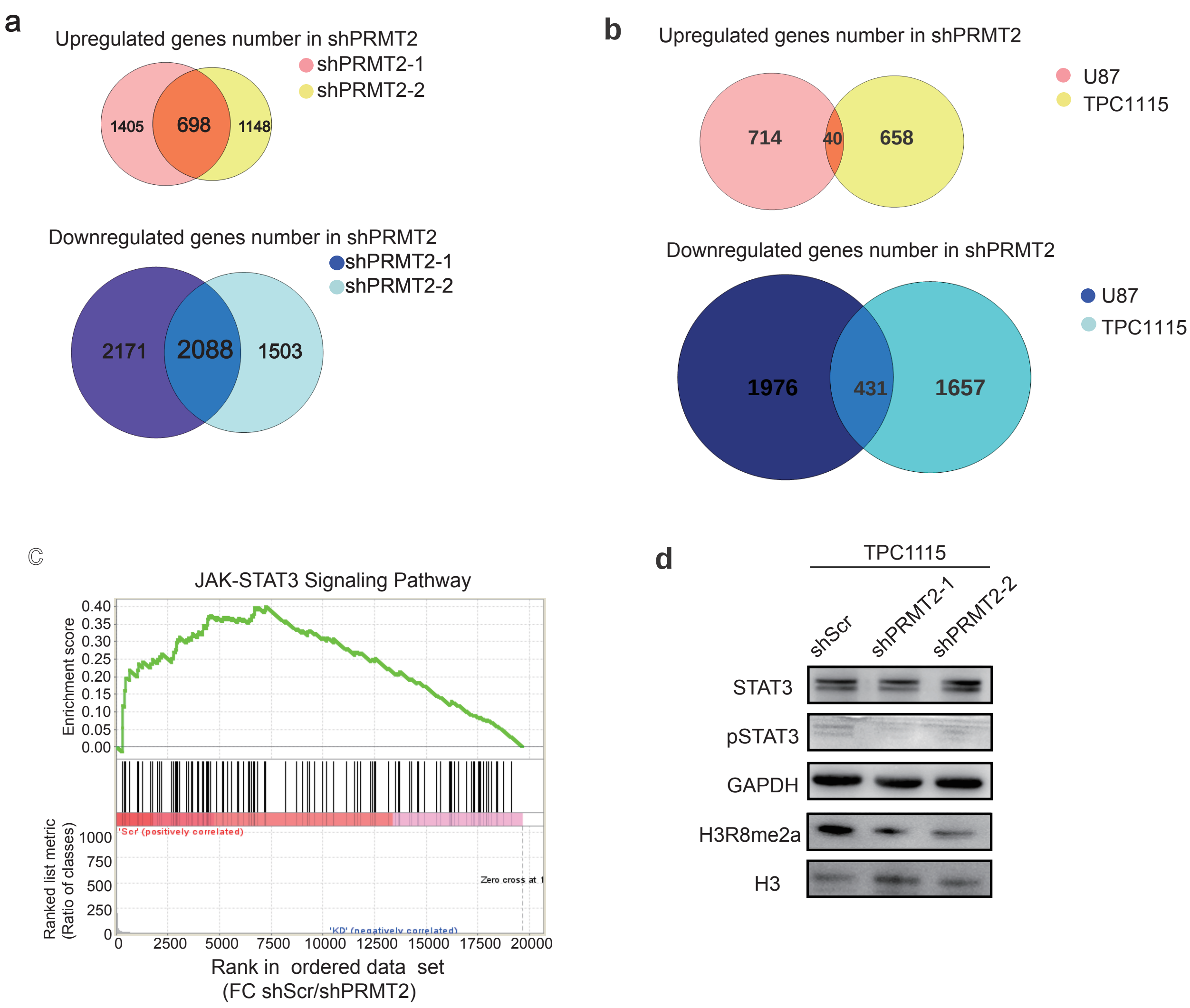

Supplementary Fig. 7 The effects of PRMT2 knockdown in TPC1115 cells. (**a**) Venn diagrams show 698 upreglated genes with overlap in TPC1115 shPRMT2-1 compared with shPRMT2-2 (Top panel) and 2088 downreglated genes with overlap in TPC1115 shPRMT2-1 compared with shPRMT2-2 (Bottom panel). (**b**) Venn diagrams show 40 upreglated genes with overlap in U87 shPRMT2 compared with TPC1115 shPRMT2 (Top panel) and 431 downreglated genes with overlap in U87 shPRMT2 compared with TPC1115 shPRMT2 (Bottom panel). (**c**) GSEA analysis shows that JAK-STAT signaling pathway was significantly affected by PRMT2 knockdown in TPC1115. (**d**) Western blotting assays analyses the effects of PRMT2 knockdown on JAK-STAT pathway in TPC1115. GAPDH and H3 were used as loading controls.

Supplementary Fig.8

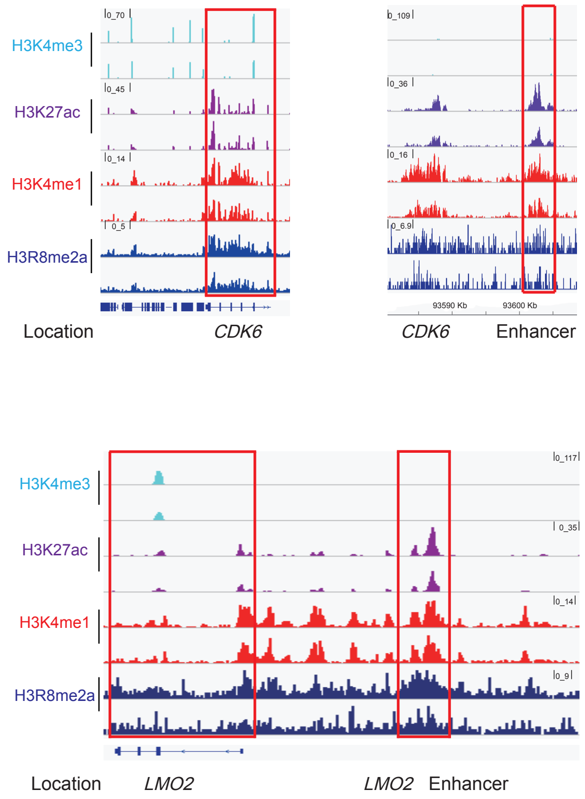

Supplementary Fig. 8 Genomic snapshots of ChIP-seq analyses of representative genes in control and PRMT2 knockdown U87 cells.

Supplementary Fig.9

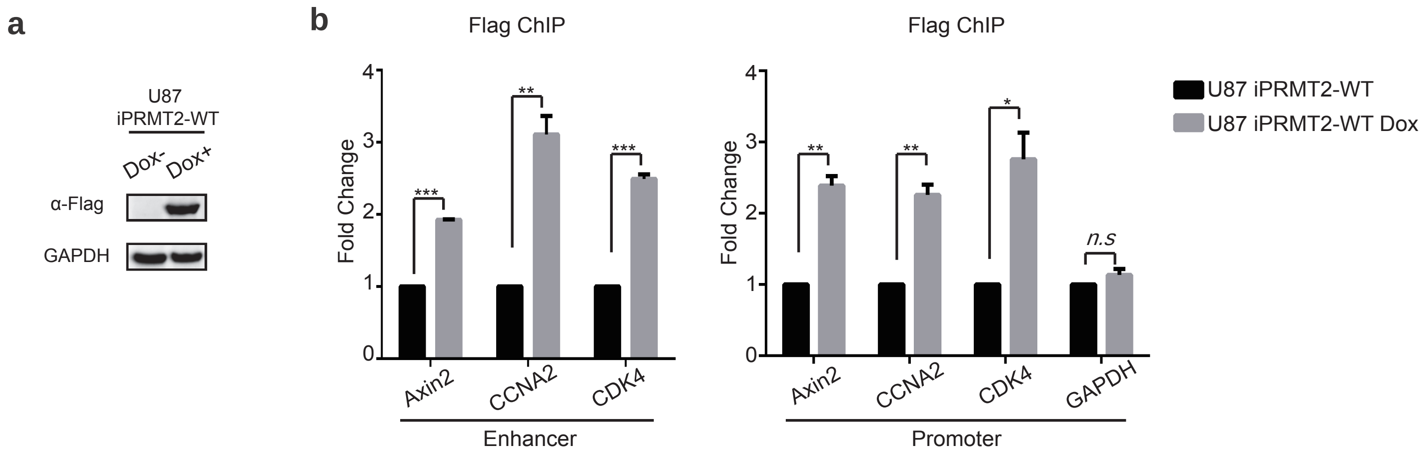

Supplementary Fig. 9 ChIP-qPCR indicated PRMT2 can bind the Promoter and Enhancer regions of *AXIN2*, *CCNA2* and *CDK4* genes. Western blot analysis indicated the induced PRMT2-flag expression.Significance level was determined using Student's two-sided *t*-tests. *$p \leq 0.05$, **$p \leq 0.01$ and ***$p \leq 0.001$.

Supplementary Fig.10

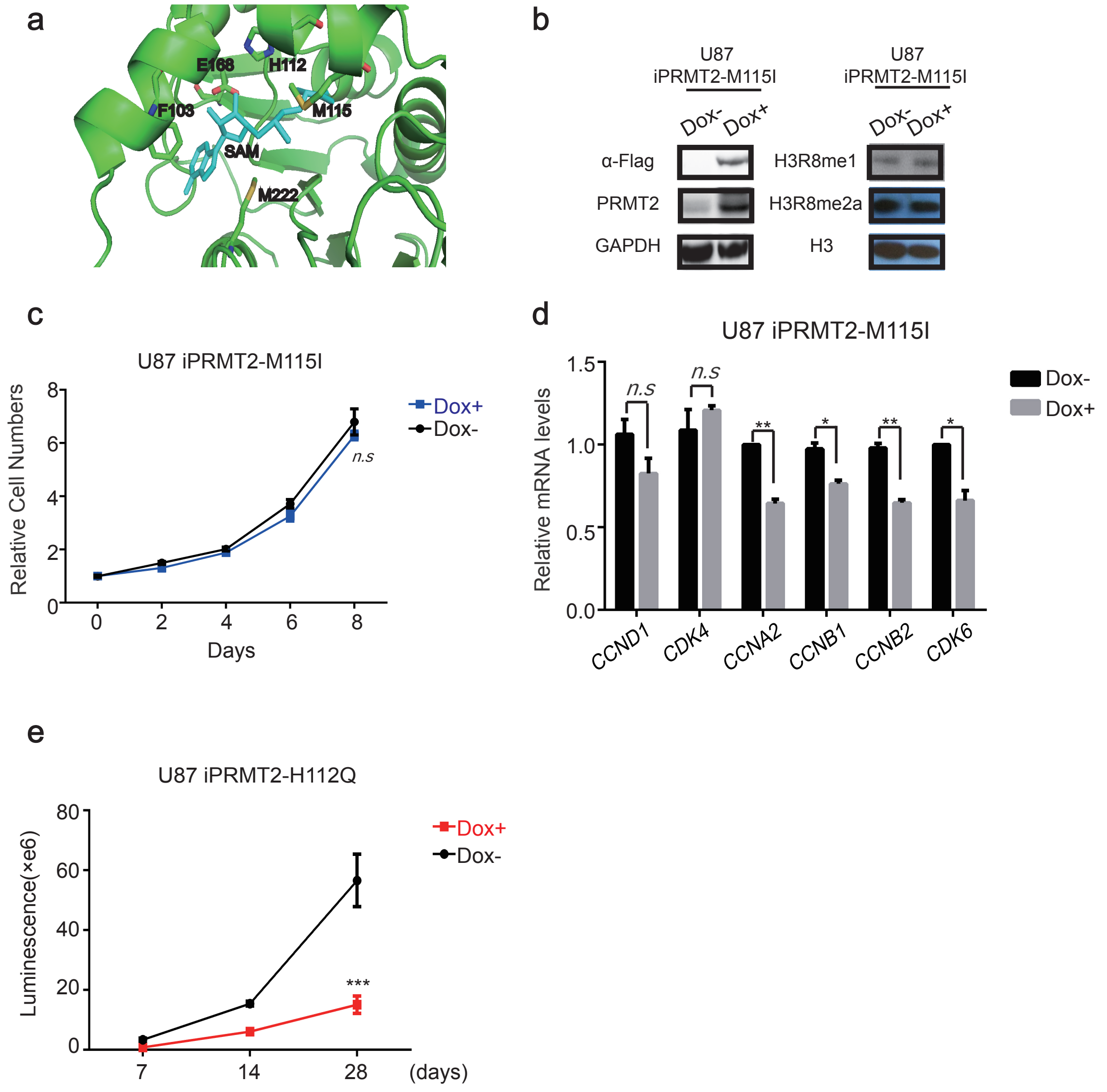

Supplementary Fig. 10 Inducible expression of PRMT2-M115I does not affect H3R8me2a levels and protumorgenic functions. (**a**) Crystal structure of PRMT2 with enlarged residues critical for binding to SAM. (**b**) Cellular levels of PRMT2 and H3R8me2a in U87 and T98G cells with or without PRMT2-M115I expression. (**c**) Cell growth curves of U87 cells with or without PRMT2-M115I expression. Error bars, ±SD, *n*=3. Significance level was determined using Student's two-sided t-tests. (**d**) RT-qPCR analyses of cell cycle associated genes in U87 cells with or without PRMT2-M115I expression.Significance level was determined using Student's two-sided t-tests. $*p \le 0.05$ and $**p \le 0.01$. (**e**) The average signals of luciferase activity in implanted mice brains at different time points (mean±SD; *n*=6). Significance level was determined using Student's two-sided *t*-tests. $***p \le 0.001$.

Supplementary Fig.11

a

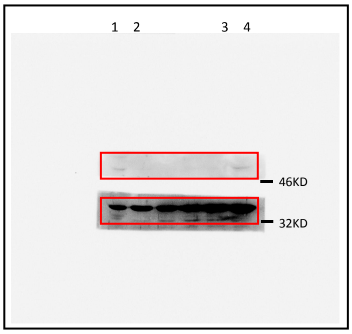

PTEN (54KD) GAPDH (36KD)

1.T98G 2.U87 3.TPC1115 4.TPC0411

c

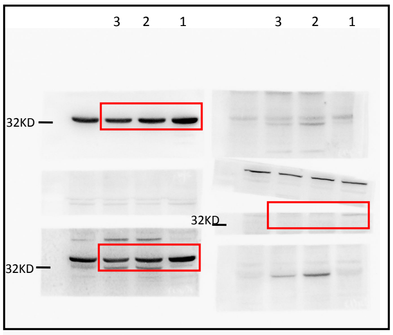

GAPDH(36KD) CCND1 (36KD) CDK6(36KD)

1.U87 shScr 2.U87 shPRMT2-1 3.U87 shPRMT2-2

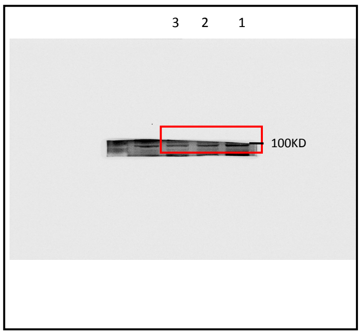

AXIN2(98KD)

1.U87 shScr 2.U87 shPRMT2-1 3.U87 shPRMT2-2

b

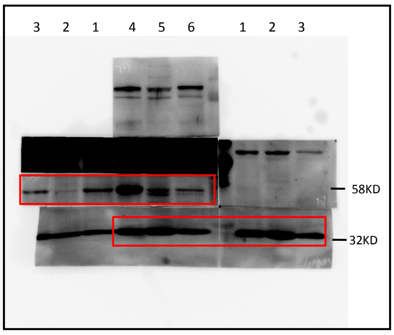

PRMT2 (49KD) GAPDH (36KD)

1.T98G shScr 2.T98G shPRMT2-1 3.T98G shPRMT2-2

4.U87 shScr 5.U87 shPRMT2-1 6.U87 shPRMT2-2

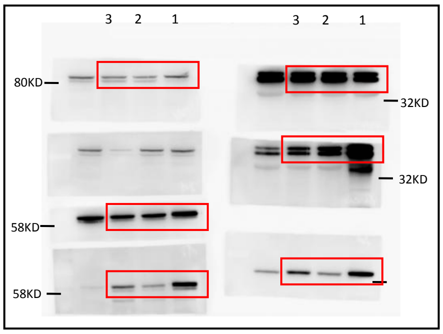

STAT3(79/86KD) AKT(60KD)

MAPK (42/44KD) pMAPK (42/44KD)

pAKT(308)(60KD) pAKT(473)(60KD)

1.U87 shScr 2.U87 shPRMT2-1 3.U87 shPRMT2-2

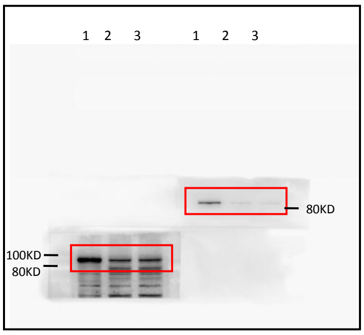

pSTAT3(79/86KD)/ β -catenin(92KD)

1.U87 shScr 2.U87 shPRMT2-1

3.U87 shPRMT2-2

d

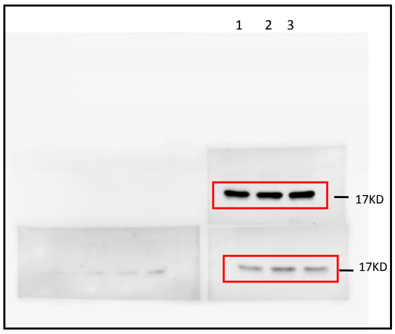

H3K4me1/H3K27ac (17KD)

1.U87 shScr 2.U87 shPRMT2-1

3.U87 shPRMT2-2

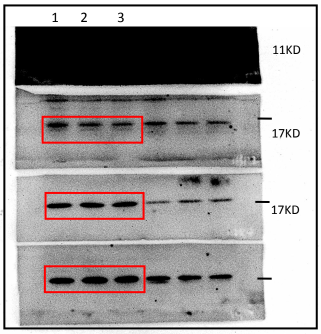

H4R3me2as (11KD)

H3k4me3/H3K9me3(17KD)

1.U87 shScr 2.U87 shPRMT2-1

3 U87 shPRMT2-2

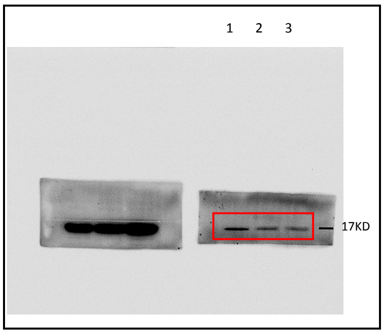

H3R8me2a (17KD)

1.U87 shScr 2.U87 shPRMT2-1 3.U87 shPRMT2-2

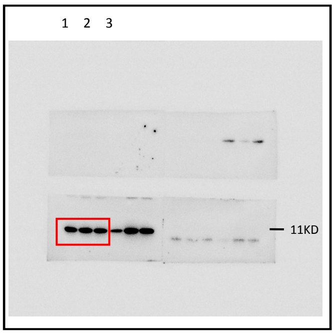

H4R3me2s (11KD)

1.U87 shScr 2.U87 shPRMT2-1

3.U87 shPRMT2-2

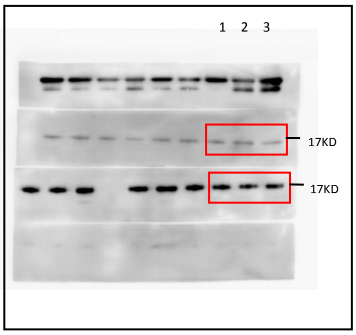

H3R8me1(17KD)/H3K27me3(17KD)

1.U87 shScr 2.U87 shPRMT2-1

3.U87 shPRMT2-2

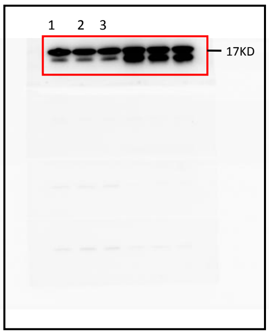

H3 (17KD)

1.U87 shScr 2.U87 shPRMT2-1

3.U87 shPRMT2-2

Supplementary Fig.12

a

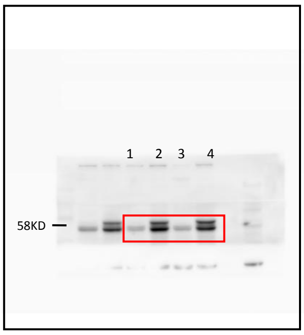

PRMT2(49KD)

1.U87 iPRMT2(H112Q) DOX-

2.U87 iPRMT2(H112Q) DOX+

3.U87 iPRMT2(M115I) DOX-

4.U87 iPRMT2(M115I) DOX+

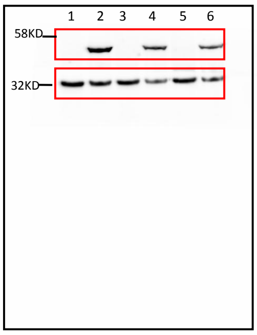

Flag(49KD)
GAPDH(37KD)

1.U87 iPRMT2 WT DOX-

2.U87 iPRMT2 WT DOX+

3.U87 iPRMT2(H112Q) DOX-

4. U87 iPRMT2(H112Q) DOX+

5.U87 iPRMT2(M115I) DOX-

6.U87 iPRMT2(M115I) DOX+

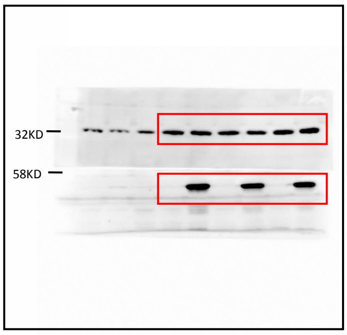

GAPDH(36KD) PRMT2(49KD)

1.T98G iPRMT2(H112Q) DOX-

2. T98G iPRMT2(H112Q) DOX+

3.T98G iPRMT2(M115I) DOX-

4.T98G iPRMT2(M115I) DOX+

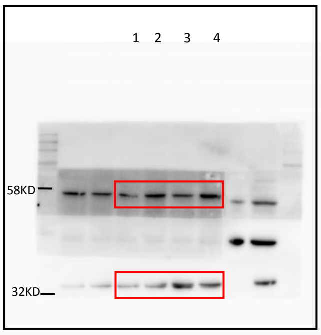

Flag(49KD)/GAPDH(36KD)

1.T98G iPRMT2(H112Q) DOX-

2. T98G iPRMT2(H112Q) DOX+

3.T98G iPRMT2(M115I) DOX-

4.T98G iPRMT2(M115I) DOX+

b

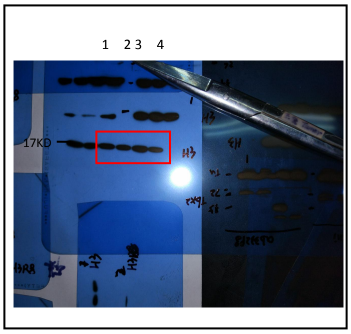

H3(17KD)

1.U87 iPRMT2(H112Q) DOX-

2. U87 iPRMT2(H112Q) DOX+

3.U87 iPRMT2(M115I) DOX-

4.U87 iPRMT2(M115I) DOX+

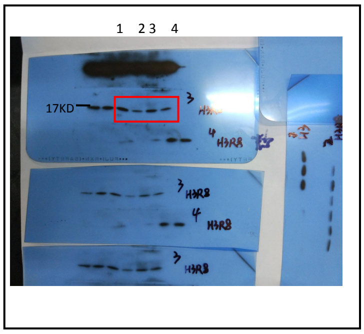

H3R8me2a(17KD)

1.U87 iPRMT2(H112Q) DOX-

2. U87 iPRMT2(H112Q) DOX+

3.U87 iPRMT2(M115I) DOX-

4.U87 iPRMT2(M115I) DOX+

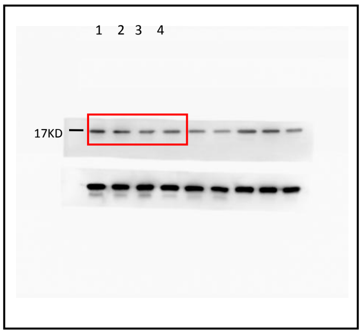

H3R8me1(17KD)

1.T98G iPRMT2(H112Q) DOX-

2. T98G iPRMT2(H112Q) DOX+

3.U87 iPRMT2(H112Q) DOX

4.U87 iPRMT2(H112Q) DOX+

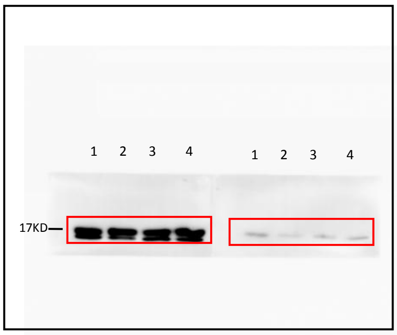

H3(17KD)/H3Rme2a(17KD)

1.T98G iPRMT2(H112Q) DOX-

2. T98G iPRMT2(H112Q) DOX+

3.T98G iPRMT2(M115I) DOX-

4.T98G iPRMT2(M115I) DOX+

c

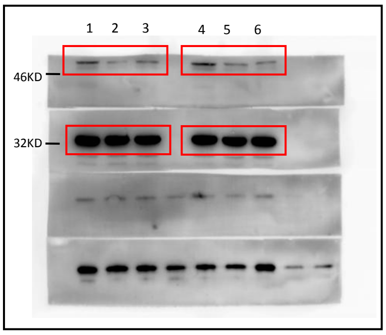

PRMT2 (49KD) GAPDH(37KD)

1.TPC1115 shScr 2.TPC11115 shPRMT2-1
3.TPC1115 shPRMT2-2 4.TPC0411 shScr
5.TPC0411 shPRMT2-1 6.TPC0411 shPRMT2-2

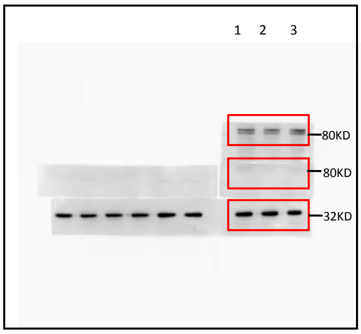

STAT3(79/86KD) pSTAT3(79/86KD)

GAPDH(37KD)

1.TPC1115 shScr 2.TPC11115 shPRMT2-1

3.TPC1115 shPRMT2-2

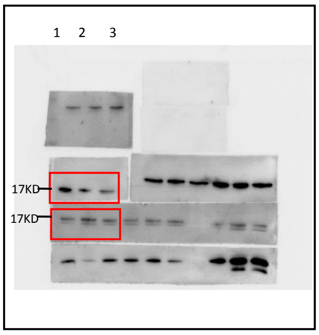

H3R8me1(17KD) H3(17KD)

1.TPC1115 shScr 2.TPC11115 shPRMT2-1

3.TPC1115 shPRMT2-2

Supplementary Fig. 11 Uncropped scans of Western blots for Fig.1 to Fig.5. (**a**) Uncropped scans of Western blots for Fig.1e. (**b**) Uncropped scans of Western blots for Fig.2b (and Supplementary Fig.4a). (**c**) Uncropped scans of Western blots for Fig.4f. (**d**) Uncropped scans of Western blots for Fig.5a.

Supplementary Fig. 12 Uncropped scans of Western blots for Fig.7 and Supplementary Fig. (**a**) and (**b**) Uncropped scans of Western blots for Fig.7c ,Supplementary Fig.9a and Supplementary Fig.10b. (**c**) Uncropped scans of Western blots for Supplementary Fig.4b and Supplementary Fig.7d.

Supplementary Table 1. Clinical information on the 21 glioma patients

Patient samples	Sex	Age (yrs)	Laterality	Grade	survival time (months)
1	F	48	Right	III	13
2	F	32	Right	IV	12
3	M	53	Right	III	26
4	M	35	Left	III	18
5	M	26	Left	II	24
6	F	49	Right	II	28
7	F	64	Right	III	3
9	M	44	Left	III	12
10	F	44	Right	IV	5
11	M	24	Right	IV	12
12	F	64	Right	IV	7
13	F	37	Left	II	12
14	F	47	Right	III	6
15	F	41	Right	III	10
16	F	76	Right	II	8
17	F	26	Right	III	8
18	F	38	Right	IV	6
19	M	49	Right	III	6
20	M	65	Left	III	7
23	F	54	Left	III	8
24	F	38	Left	II	6

M: male
F: female

Supplementary Table 2. Primer sequences and antibodies

	shRNA Oligonucleotides
Non-targeting control	5′-GAAUACGUACCCCAUUAUA-3′
PRMT1	5′-GTGTTCCAGTATCTCTGATTA-3′
PRMT2 #1	5′-CCAGCCACGAACAACTAAATA-3′
PRMT2 #2	5′-GAACGGCTTTGCTGACATCAT-3′
PRMT3	5′-CCTTGGGAGAAAGAAGAGTAT-3′
PRMT4	5′-CTATGACTTGAGCAGTGTTAT-3′
PRMT5	5′-GCCCAGTTTGAGATGCCTTAT-3′
PRMT6	5′-CACCGGCATTCTGAGCATCTT-3′
PRMT7	5′-GCCTCAAGAGATCCTGACTTT-3′
PRMT8	5′-GCTATCGTTCACATCTGCATT-3′
PRMT9	5′-GCTGGCACTTTATCATGCTTA-3′

Primers used for qPCR

PRMT1	forward: 5′-GCGGTGAAGATCGTCAAAGC-3′
	reverse: 5′-GACTCGTAGAAGAGGCAGTA-3′
PRMT2	forward: 5′-TCTCTTCTGTGCACACTATG-3′
	reverse: 5′-GGCAGCACCACATCCTCCAC-3′
PRMT3	forward: 5′-CTTCGCCCCAGCTTTA-3′
	reverse: 5′-TACGGGCATTATGGGAT-3′
PRMT4	forward: 5′-GATGAACAGCTCTACATGGA-3′
	reverse: 5′-AGGCTGCCGGAAATACTCAT-3′
PRMT5	forward: 5′-GAAAAGGACCCCATCAAATA-3′
	reverse: 5′-ATTGGTATCCTTCTCCTCTT-3′
PRMT6	forward: 5′-TCCTGCCGGGACCAGTGGAG-3′
	reverse: 5′-ACCGCCCTCCTTCAGCCACT-3′
PRMT7	forward: 5′-ACCTGCTCTGGAACCGGCCT-3′
	reverse: 5′-GGTGATGGGCCAGCACGGAG-3′
PRMT8	forward: 5′-GTTTCCAGACCGGGCAGCTT-3′
	reverse: 5′-TCCACGATGTCCACTAGAGG-3′
PRMT9	forward: 5′-TGCTGATGTTTGGGTTGCTT-3′
	reverse: 5′-CGTTCTGTTCCTTGAACAGC-3′
AXIN2	forward: 5′-AGTGTGAGGTCCACGGAAAC-3′
	reverse: 5′-CTTCACACTGCGATGCATTT-3′
CCND1	forward: 5′-CCGTCCATGCGGAAGATC-3′
	reverse: 5′-ATGGCCAGCGGGAAGAC-3′
CCNA2	forward: 5′-CCTCCTTGGAAAGCAAACAGT-3′
	reverse: 5′-CAGGGCATCTTCACGCTCTAT-3′
CCNB1	forward: 5′-TGCTTATCTGAGACAACTTGAGGA-3′
	reverse: 5′-ACCAATTTCTGGAGGGTACATTT-3′
CCNB2	forward: 5′-CAACCCACCAAAACAACAAATG-3′
	reverse: 5′-CAATCTTCGTTATCAATGTCCTCG-3′
CDK4	forward: 5′-CCATCAGCACAGTTCGTGAGGT-3′
	reverse: 5′-TCAGTTCGGGATGTGGCACAGA-3′
CDK6	forward: 5′-TGGTCAGGTTGTTTGATGTGTG-3′
	reverse: 5′-GAAGTCAGCGAGTTTTATTTGTCC-3′
TIAM1	forward: 5′-AAGACGTACTCAGGCCATGTCC-3′
	reverse: 5′-GACCCAAATGTCGCAGTCAG-3′

DKK3	forward: 5′-TCATCAGAAGTGAACCTGGCAA-3′
	reverse: 5′-CTTCTGCCTTCTTCGTCTCCC-3′
GAPDH	forward: 5′-GCACCGTCAAGGCTGAGAAC-3′
	reverse: 5′-TGGTGAAGACGCCAGTGGA-3′
RPO	forward: 5′-TTCATTGTGGGAGCAGAC-3′
	reverse: 5′-CAGCAGTTTCTCCAGAGC-3′
AXIN2 promoter	forward: 5′-CTGGAGCCGGCTGCGCTTTGATAA-3′
	reverse: 5′-CGGCCCCGAAATCCATCGCTCTGA-3′
CCNA2 promoter	forward: 5′-CAGGGTCCCGCGCTGCTC-3′
	reverse: 5′-TGGCGCCAGTTTTTCGGGGT-3′
CDK4 promoter	forward: 5′-CACACTGGAAGCAAGCACTCAG-3′
	reverse: 5′-TGATGCTGTCACCTTTTCGCT-3′
AXIN2 enhancer	forward: 5′- AGTAAGCAACCCTCACCTGC-3′
	reverse: 5′-ACTCACTCTTCCCCCAAAGC-3′
CCNA2 enhancer	forward: 5′-TCTCCTTGTCCACAGATAATGC-3′
	reverse: 5′-TCAACACCGACCTTACTTTCAT-3′
CDK4 enhancer	forward: 5′-GGTTGGTTCTGGGTTAGGC-3′
	reverse: 5′-AGCCTGCTTCCCTCATTGTC-3′
GAPDH promoter	forward: 5′-GCCACATCGCTCAGACAC-3′
	reverse: 5′-CATACGACTGCAAAGACCC-3′

Primers used for cloning and mutation

PRMT2 cloning	forward: 5′-ATGGCAACATCAGGTGACTG-3′
	reverse: 5′-TCATCTCCAGATGGGGAAGA-3′
PRMT2(H112Q)	forward: 5′-GAAACTCCAGTTGGAGATGTTGGCAGACCA-3′
	everse: 5′-CTGGAGTTTCAGAGTTCCATAGCTGCCGAAG-3′
PRMT2(M115I)	forward: 5′-CTTGGAGATATTGGCAGACCAGCCACGAAC-3′
	everse: 5′-GGTCTGCCAATATCTCCAAGTGGAGTTTCAG-3′

Antibodies	Source	Identifier	Application	Concentration used
PRMT2	LifeSpan BioSciences	Cat#L482512	IB	1:1000
PRMT2	Santa Cruz Biotechnology	Sc-393254	IF,IHC	1:100,1:100
GAPDH	Abways	Cat#ab0037	IB	1:5000
Histone H3	Millipore	Cat#07-690	IB	1:4000
AKT(pan)	Cell Signaling Technology	Cat#4691	IB	1:1000
p-AKT(Thr308)	Cell Signaling Technology	Cat#13038	IB	1:1000
p-AKT(Ser473)	Cell Signaling Technology	Cat#4060	IB	1:1000
MAPK	Cell Signaling Technology	Cat#4695	IB	1:1000
p-MAPK(Thr202/Tyr204)	Cell Signaling Technology	Cat#4370	IB	1:1000
STAT3	Abways	Cat#CY5165	IB	1:1000
p-STAT3	Abways	Cat#CY6566	IB	1:1000
AXIN2	Absin	Cat#abs125590a	IB	1:500
CCND1	Absin	Cat#abs130610	IB,IHC	1:1000,1:100
CDK6	Wanleibio	Cat#wl01676a	IB	1:1000
CDK4	Wanleibio	Cat#wl02274	IHC	1:200
CCNB1	Santa Cruz Biotechnology	SC-245	IB,IHC	1:1000,1:200
β-catenin	Abcam	Cat#ab32572	IB	1:1000
α-Flag	Sigma-Aldrich	Cat#F7425	IB,ChIP	1:2000
PTEN	Cell Signaling Technology	Cat#9188	IB	1:1000
H3K4me1	Cell Signaling Technology	Cat#5326	IB,ChIP	1:1000
H3K4me3	Cell Signaling Technology	Cat#9751	IB,ChIP	1:1000
H3K27me3	Cell Signaling Technology	Cat#9733	IB,ChIP	1:1000
H3K27ac	Abcam	Cat#ab45173	IB,ChIP	1:1000
H3R8me2a	Novus Biologicals	Cat#NB21-1062	IB,IF,IHC,ChIP	1:1000,1:500,1:500
H3K9me3	Cell Signaling Technology	Cat#13969	IB,ChIP	1:1000
H4R3me2a	PTM-Biolab	Cat#PTM-667	IB	1:1000
H4R3me2s	PTM-Biolab	Cat#PTM-639	IB	1:1000
H3R8me1	PTM-Biolab	Cat#PTM-686	IB	1:1000
Normal rabbit IgG antibody	Cell Signaling Technology	Cat#2729	ChIP	
Anti-rabbit IgG, HRP-linked	Cell Signaling Technology	Cat#7074	IB	1:4000
Anti-mouse IgG, HRP-linked	Cell Signaling Technology	Cat#7076	IB	1:3000
Anti-rabbit IgG, FITC-linked	ZSBIO	15176-1-AP	IF	1:200
