# Supplementary material for: PRMT2 links histone H3R8 asymmetric dimethylation to oncogenic activation and tumorigenesis of glioblastoma
Source: Nat Commun. 2018 Oct 31;9:4552. doi: 10.1038/s41467-018-06968-7 (PMC6208368; doi:10.1038/s41467-018-06968-7)
Supplement: Supplementary file 3 — Description of Additional Supplementary Files [file 41467_2018_6968_MOESM3_ESM.pdf]

Description of Additional Supplementary Files

File Name: Supplementary Data 1
Description: The differentially expressed genes (DEGs) in shPRMT2-1 and shPRMT2 compared with shScr (fold change≥2 and FDR≤0.05) in U87.

File Name: Supplementary Data 2
Description: Differentially expressed genes in shPRMT2-1 compared with shScr in TPC1115 (fold change≥2 and FDR≤0.05).
